# The Rho GTPase Cdc42 regulates hair cell planar polarity and cellular patterning in the developing cochlea

**DOI:** 10.1242/bio.20149753

**Published:** 2015-03-13

**Authors:** Anna Kirjavainen, Maarja Laos, Tommi Anttonen, Ulla Pirvola

**Affiliations:** Department of Biosciences, Viikinkaari 1, 00014 University of Helsinki, Finland

**Keywords:** Planar polarity, Patterning, Development, Hair cell, Stereociliary bundle, Kinocilium, Microtubules, aPKC, Auditory

## Abstract

Hair cells of the organ of Corti (OC) of the cochlea exhibit distinct planar polarity, both at the tissue and cellular level. Planar polarity at tissue level is manifested as uniform orientation of the hair cell stereociliary bundles. Hair cell intrinsic polarity is defined as structural hair bundle asymmetry; positioning of the kinocilium/basal body complex at the vertex of the V-shaped bundle. Consistent with strong apical polarity, the hair cell apex displays prominent actin and microtubule cytoskeletons. The Rho GTPase Cdc42 regulates cytoskeletal dynamics and polarization of various cell types, and, thus, serves as a candidate regulator of hair cell polarity. We have here induced *Cdc42* inactivation in the late-embryonic OC. We show the role of Cdc42 in the establishment of planar polarity of hair cells and in cellular patterning. Abnormal planar polarity was displayed as disturbances in hair bundle orientation and morphology and in kinocilium/basal body positioning. These defects were accompanied by a disorganized cell-surface microtubule network. Atypical protein kinase C (aPKC), a putative Cdc42 effector, colocalized with Cdc42 at the hair cell apex, and aPKC expression was altered upon Cdc42 depletion. Our data suggest that Cdc42 together with aPKC is part of the machinery establishing hair cell planar polarity and that Cdc42 acts on polarity through the cell-surface microtubule network. The data also suggest that defects in apical polarization are influenced by disturbed cellular patterning in the OC. In addition, our data demonstrates that Cdc42 is required for stereociliogenesis in the immature cochlea.

## INTRODUCTION

The mammalian auditory sensory epithelium, the organ of Corti (OC) of the cochlea, consists of sensory hair cells and supporting cells. These cells are characterized by unique morphologies, specializations of their actin and microtubule cytoskeletons and complexity in cytoarchitectural organization as an epithelium (Raphael and Altschuler, 2003). One row of inner hair cells (IHCs) and three rows of outer hair cells (OHCs) together with interdigitated supporting cells form a checkerboard pattern, created by Notch signaling-mediated lateral inhibition (Eddison et al., 2000) and convergent extension (CE)-based cellular movements that are supported by cell-adhesive molecules (McKenzie et al., 2004; Wang et al., 2005; Togashi et al., 2011).

The mechanotransduction organelle at the hair cell's apical surface, the stereociliary bundle, comprises rows of filamentous actin (F-actin)-rich stereocilia arranged in graded heights into a V-shaped structure. The vertex of the developing bundle is marked by the presence of a specialized primary cilium, the kinocilium, that is crucial for determining the orientation and morphology of the bundle (Ross et al., 2005; Jones et al., 2008; Cui et al., 2011; Sipe and Lu, 2011). The uniform orientation of hair bundles at the surface of the OC – the vertex of each bundle pointing laterally – defines planar cell polarity (PCP) at tissue level. In addition, hair cells show planar polarity at subcellular level, manifested as normally oriented bundles coupled with asymmetric bundle morphology, i.e. positioning of the kinocilium at the vertex, behind the longest stereocilia. We refer to hair cell intrinsic polarity in this case. In the OC of the mouse, planar polarity and cellular organization are established during late-embryogenesis. Structural maturation during the early postnatal life reinforces the polarized cellular structures, an event that is essential for proper sound perception (Forge et al., 1997).

The non-canonical Wnt/PCP pathway is required for the establishment of PCP in the OC, as evidenced by random hair bundle orientation in mutant mice in which the core components of this pathway are inactivated (Curtin et al., 2003; Montcouquiol et al., 2003; Wang et al., 2006). Much less is known about the molecules underlying the establishment of hair cell intrinsic polarity, a process that can function downstream or parallel of the core PCP pathway. Also, molecular interactions between the core PCP proteins and the regulators of intrinsic hair cell polarity are poorly understood. Recent studies suggest that, in addition to the mechanisms operating at the hair cell apex (Sipe and Lu, 2011; Sipe et al., 2013; Ezan et al., 2013; Tarchini et al., 2013), hair cell intrinsic polarity is influenced by mechanisms originating from the junctions between hair cells and supporting cells (Fukuda et al., 2014).

In non-mammalian vertebrates, members of the Rho GTPase family mediate cell polarization through regulation of cytoskeletal reorganization, formation of junctional complexes, and activation and localization of polarity proteins (Schlessinger et al., 2009). In the developing OC of the mouse, the Rho GTPase Rac1 has been shown to regulate apical polarity of hair cells (Grimsley-Myers et al., 2009). We have previously shown that another Rho GTPase, Cdc42, is required for the development of apical polarity of supporting cells of the OC during the early postnatal life (Anttonen et al., 2012). In the present study, we postulate that Cdc42 regulates apical polarization of auditory hair cells as well. As hair cell polarity is established during late-embryogenesis, we concentrate on this prenatal period. Interestingly, a well-known effector of Cdc42, the atypical protein kinase C (aPKC), has recently been shown to be involved in the establishment of apical polarity of embryonic hair cells (Ezan et al., 2013; Tarchini et al., 2013).

We show here that Cdc42 is required for cellular patterning in the OC and for the establishment of planar polarity of hair cells. In addition, we demonstrate that Cdc42 regulates stereociliogenesis in the immature cochlea.

## MATERIALS AND METHODS

### Mice

Mice homozygous for the floxed *Cdc42* allele (*Cdc42^loxP/loxP^*) were crossed with mice carrying the *Fgfr3-iCre-ER^T2^* transgene to obtain *Cdc42^loxP/loxP^;Fgfr3-iCre-ER^T2^* animals. In the *Cdc42^loxP^* mice, exon 2 encoding guanine nucleotide binding sequence is flanked by loxP sites (Wu et al., 2006). *Cdc42^loxP/wt^;Fgfr3-iCre-ER^T2^* and *Cdc42^loxP/loxP^* mice were used as control animals. Genotyping by PCR was conducted as previously described (Wu et al., 2006; Young et al., 2010). To study the characteristics of iCre-mediated recombination *Fgfr3-iCre-ER^T2^* transgenic mice were bred with the *ROSA26tm14(CAG-tdTomato) Cre*-conditional reporter mice (obtained from the Jackson Laboratory) to generate *Fgfr3-iCre-ER^T2^;Ai14(tdTomato)* animals. The *Ai14(tdTomato)* transgene expression was detected by direct visualization of the tdTomato native fluorescence in tails of the animals. Timed pregnancies were established by the detection of vaginal plug, taken the morning of plug observation as embryonic day 0.5 (E0.5). Both females and males were used in the analysis. Mouse lines were maintained in a mixed background. All animal work has been conducted according to relevant national and international guidelines. Approval for animal experiments has been obtained from the National Animal Experiment Board.

### Induction of iCre-mediated recombination

Pregnant mice were injected intraperitoneally with 3 mg of tamoxifen (Sigma, prepared as described earlier; Anttonen et al., 2012) at E13.5 and E14.5 or at E15.5 and E16.5. Dams were sacrificed and embryos dissected at E18.5. For postnatal studies, E18.5 embryos were surgically delivered and transferred to foster mothers. Pups were killed at postnatal day 6 (P6) and P18.

### Whole mount specimens

For confocal microscopy, inner ears were dissected from E18.5 embryos and fixed in 4% paraformaldehyde (PFA) in PBS for 5 h. P6 and P18 cochleas were perilymphatically fixed with PFA before immersion in this fixative. For Cdc42 antibody staining, dissected inner ears were fixed in ice-cold 10% trichloroacetic acid (TCA) for 1 h. For immunofluorescence, whole mounts were blocked for 30 min with 10% normal serum in PBS containing 0.25% Triton-X-100 (PBS-T), followed by incubation overnight at +4°C with the appropriate primary antibodies in PBS-T. The following primary antibodies were used: rabbit monoclonal acetylated tubulin (Cell Signaling Technology); mouse monoclonal acetylated tubulin; rabbit polyclonal gamma-tubulin; rat monoclonal E-cadherin antibody (all from Sigma); rat monoclonal nectin 2, clone 502-57; rat monoclonal nectin 3, clone 103-A1 (both from Abcam); mouse monoclonal ZO-1 (Molecular Probes/Invitrogen); mouse monoclonal β-catenin; mouse monoclonal Rab11a; mouse monoclonal Frizzled 6; mouse monoclonal Cdc42 (all from BD Biosciences); mouse monoclonal aPKCζ; rabbit polyclonal aPKCλ/ι; rabbit polyclonal phospho-PKCζ/λ; mouse monoclonal Cdc42 (all from Santa Cruz Biotechnology); rabbit polyclonal Vangl2 (Montcouquiol et al., 2006). Secondary antibodies conjugated to Alexa 488, 568, 594 or 647 were used for detection. Following antibody incubations, F-actin was visualized using Oregon green-labeled phalloidin (1:400, 20 min at room temperature). Nuclei were stained with DAPI (Sigma). ProLong Gold anti-fade reagent was used for mounting (Molecular Probes/Invitrogen). Confocal images were acquired using a Leica TCS SP5 laser scanning microscope with Plan Apochromat 63×/1.3 NA and 20×/0.7 NA glycerol objectives. The acquisition software was Leica LAS AF. Z-projections were processed with Imaris 7 (Bitplane Scientific Software). Blind 3D deconvolution was made with AutoQuant X3 (Media Cybernetics). A minimum of 5 cochleas per antibody and genotype were analyzed.

### Histological sections

Dissected inner ears from E18.5 embryos were fixed overnight in 4% PFA, embedded in paraffin and cut to 5-µm-thick sections. They were processed for antibody stainings as previously described (Anttonen et al., 2012). The following primary antibodies were used: rabbit RFP (red fluorescence protein, Rockland Immunochemicals); rabbit monoclonal cleaved caspase-3 (Cell Signaling Technology); rabbit polyclonal myosin 6 (Hasson et al., 1997); guinea pig polyclonal Gfi1 (Wallis et al., 2003); rabbit monoclonal Ki-67 (LabVision/ThermoScientific); mouse monoclonal p27^Kip1^ (BD Biosciences); goat polyclonal Prox1 (R&D Systems); mouse monoclonal fodrin (Ylikoski et al., 1992); mouse monoclonal Rab11. Detection was done with the Vectastain Elite ABC kit or Vectastain Mouse-On-Mouse kit and the diaminobenzidine substrate kit (all from Vector Laboratories). Sections were counterstained with 3% methyl green and mounted in Permount (Fisher Scientific).

In situ hybridization was performed with [35^S^]-labeled *Cdc42* (Cappello et al., 2006) and *Fgfr3* (Peters et al., 1993) riboprobes on PFA-fixed paraffin sections. Hematoxylin was used for counterstaining. Sections were analyzed with a BX61 microscope (Olympus) using bright- and darkfield optics. Images were acquired through the DP70 CCD color camera and cell'F software (Olympus) and processed using Adobe Photoshop CS6 (Adobe Systems). For visualization of in situ hybridization signals, autoradiographic silver grains in the darkfield image were selected, colored red and superimposed onto the brightfield image. For all antibodies and probes used in paraffin sections, a minimum of 4 cochleas per genotype were prepared for histological analysis.

### Serial block-face scanning electron microscopy

Specimens were prepared and processed for serial block-face scanning electron microscopy (SBEM) and data was analyzed as previously described (Anttonen et al., 2014).

### Hair bundle orientation

To measure the hair bundle orientation, a line was drawn from the place of the kinocilium through the center of the bundle (bisecting line). Another line was drawn parallel to the medio-lateral (planar polarity) axis. The angle of bundle orientation was defined as the angle formed between these lines. In control animals it is close to 0°. Oriana3 plots were drawn from the orientations of OHCs (OHC1-OHC3). The orientation was divided into 5° sections, each bar showing cell numbers within that 5° section. A total number of 119 and 88 OHCs from the medial coil of mutant (n = 3) and controls (n = 3) cochleas, respectively, were used for analysis.

### Correlation between hair cell shape and bundle orientation

In the medial coil of the cochlea, OHC diameters were measured at the level below adherens junctions, and stereociliary bundle orientations defined as described in the previous paragraph, using the Image-Pro Plus software (Media Cybernetics) (n = 76 OHCs, 4 mutant cochleas; n = 21 OHCs, 2 control cochleas). Regression analysis was performed using the IBM SPSS Statistics and Microsoft Excel softwares.

## RESULTS

### *Fgfr3-iCre-ER^T2^* drives efficient recombination in the embryonic organ of Corti

**Fig. 1.**
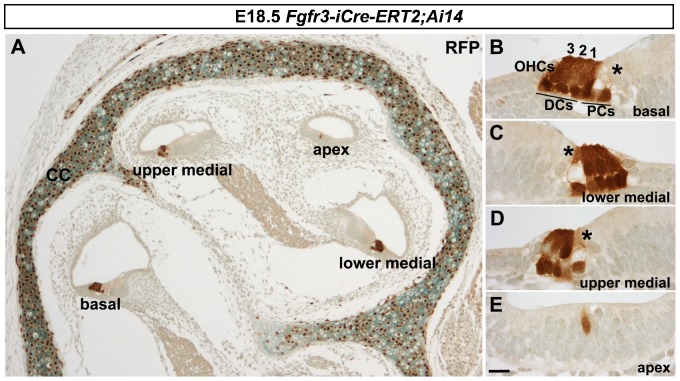
Recombination pattern obtained with the *Fgfr3-iCre-ER^T2^* mice. (A–E) *Fgfr3-iCre-ER^T2^;Ai14(tdTomato)* mice treated with tamoxifen at E13.5 and E14.5 show recombination in the organ of Corti and in the cochlear capsule, revealed by red fluorescence protein (RFP) immunohistochemistry on paraffin sections at E18.5. Recombination follows a base-to-apex gradient along the length of the cochlear duct. All Deiters' and pillar cells and OHCs in the basal (B) and lower medial coils (C) are recombined. A large part of these cells are recombined in the upper medial coil (D), but only rare recombined cells are found in the apex (E). Inner hair cells (asterisks in B–D) are not stained for RFP. The three OHC rows are numbered. Abbreviations: OHCs, outer hair cells; PCs, pillar cells; DCs, Deiters' cells; CC, cochlear capsule. Scale bar shown in E: A, 25 µm; B–E, 7 µm.

Cdc42 is widely expressed and has several roles in developing cells (Melendez et al., 2011). Consistently, *Cdc42* knockout mice show early-embryonic lethality (Chen et al., 2000). As we wished to reveal the role of Cdc42 during cellular differentiation in the late-embryonic OC, a conditional and inducible approach was required to bypass the likely effects of Cdc42 on dividing progenitor cells of the early otocyst. We used the *Fgfr3-iCre-ER^T2^* transgenic mouse line (Young et al., 2010) that was crossed with the *Cdc42^loxP/loxP^* mutant mice (Wu et al., 2006). We have previously shown that *Fgfr3-iCre-ER^T2^* mice can be used for inducible, supporting cell-specific gene inactivation in the postnatal OC (Anttonen et al., 2012). To study recombination characteristics in the embryonic cochlea, *Fgfr3-iCre-ER^T2^* mice were crossed with the *ROSA26tm14(CAG-tdTomato)* reporter mice to generate *Fgfr3-iCre-ER^T2^;Ai14(tdTomato)* mice. Recombined cells were recognized by RFP immunohistochemistry in paraffin-embedded cross-sections through the cochlea (Fig. 1A–E). Recombination was induced at E13.5 and E14.5, at the stages when *Fgfr3* starts to be expressed in the presumptive OC (Hayashi et al., 2010). At this age, precursor cells have exited the cell cycle and are initiating differentiation into hair cells or supporting cells (Ruben, 1967; Chen and Segil, 1999). At E18.5, basal and medial coils of the cochlea of the *Fgfr3-iCre-ER^T2^;Ai14(tdTomato)* double-transgenic mice showed recombination in OHCs and in two types of supporting cells, the Deiters' and pillar cells (Fig. 1B–D). Importantly, the whole population of these cells showed recombination in the basal and medial coils, the regions of the cochlear duct analyzed in the present study. IHCs lacked RFP signal (asterisks in Fig. 1B–D). The apical cochlear coil showed recombination only in scattered sensory epithelial cells (Fig. 1E). This recombination pattern correlates well with the cell type-specific expression of *Fgfr3* and with the dynamic *Fgfr3* expression along the base-to-apex differentiation gradient in the developing cochlear duct (Hayashi et al., 2010). We conclude that *Fgfr3-iCre-ER^T2^*-mediated recombination efficiently targets OHCs, Deiters' cells and pillar cells of the late-embryonic OC.

### Cdc42 expression in the embryonic organ of Corti

**Fig. 2.**
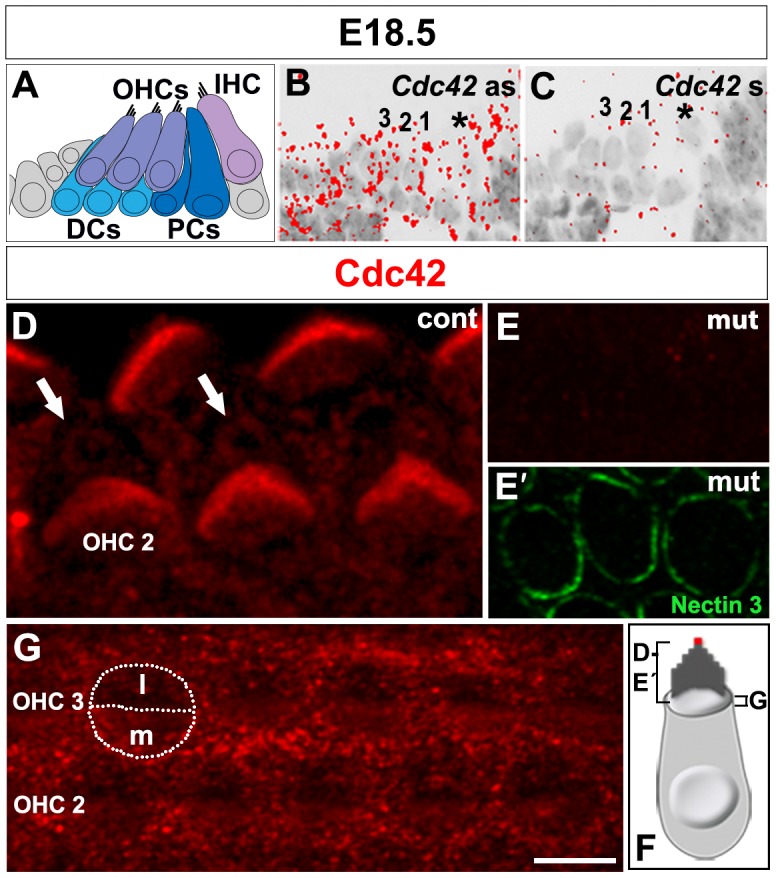
Cdc42 in situ hybridization and immunostaining in the organ of Corti at E18.5. (A) Schematic representation of the cytoarchitecture of the organ of Corti at E18.5. (B,C) The antisense (as) probe shows ubiquitous *Cdc42* mRNA expression in the sensory epithelium (B). The *Cdc42* sense (s) probe reveals the background level. Asterisk marks IHC. The three OHC rows are numbered. (C). (D–F) Confocal images of cochlear whole mount specimens stained for antibodies against Cdc42. Strong expression is seen in the stereocilia of hair bundles and weaker expression around centrioles (arrows), lateral to the bundle (D). Double-labeling shows the absence of Cdc42 immunostaining in the *Cdc42^loxP/loxP^;Fgfr3-iCre-ER^T2^* mice (E), while nectin 3 marks the junctions between OHCs and Deiters' cells (E′). Control animals show weak Cdc42 staining in the medial surface domain of OHCs. Contours of an OHC are outlined and a line is drawn to separate the medial (m) and lateral (l) surface domains. Staining is also found at the level of adherens junctions between OHCs and Deiters' cells (G). (F) Schematic picture shows the planes and areas covered in the immunofluorescence views. The red dot marks the site of the kinocilium and basal body. Abbreviations: IHC, inner hair cell; OHCs, outer hair cells; DCs, Deiters' cells; PCs, pillar cells. Scale bar shown in G: A–C, 20 µm; D, 3 µm; E–G, 5 µm.

To study *Cdc42* mRNA expression in the developing cochlea, we used radioactive in situ hybridization on paraffin sections. As analyzed at E13.5, E15.5, E18.5 and P0, *Cdc42* was ubiquitously expressed in the presumptive and, later, in differentiating hair cells and supporting cells (Fig. 2A,B; data not shown). Signal obtained by the *Cdc42* antisense probe clearly surpassed the background signal obtained with the *Cdc42* sense probe (Fig. 2B,C). Upon tamoxifen-induced recombination, the level of *Cdc42* antisense signal in the OC of the *Cdc42^loxP/loxP^;Fgfr3-iCre-ER^T2^* mice (hereafter termed as the mutant mice) was comparable to background signal, consistent with prior studies (data not shown; Anttonen et al., 2012).

We next used immunocytochemistry to localize Cdc42 protein in the OC at E18.5. Comparable results were obtained with two commercial antibodies in TCA-fixed whole mount specimens. Cdc42 was localized to the OHC apex (Fig. 2D–G). Expression was found in the region around the basal body from where microtubules radiate at the cell surface (Fig. 2D). Weaker expression was found in the apical cytoplasm, on the medial side of the bundle (Fig. 2G). In addition, stereocilia showed strong expression (Fig. 2D) as well as the contact sites between supporting cells and OHCs at the level of adherens junctions (Fig. 2G). Cdc42 was also expressed in the apices of IHCs (data not shown) and supporting cells (Fig. 2G). Cdc42 immunostaining was abolished from OHCs of the *Cdc42^loxP/loxP^;Fgfr3-iCre-ER^T2^* mice while the unrecombined IHCs showed maintained expression (Fig. 2E,E′; data not shown).

### *Cdc42* inactivation alters cellular patterning and cell shapes, but not global morphology of the organ of Corti

Whole mount specimens and paraffin-embedded cross-sections revealed an unaltered global morphology of the cochlea of the *Cdc42* mutant mice at E18.5, including unaltered length and width of the OC (supplementary material Fig. S1A,B). To find out whether *Cdc42* inactivation triggers cell cycle activity, expression of the proliferation marker Ki-67 and the cyclin-dependent kinase inhibitor p27^Kip1^, a negative cell cycle regulator, were studied on paraffin sections. The OC of both control and mutant mice showed strong p27^Kip1^ immunofluorescence in supporting cells and absence of Ki-67 staining in hair cells and supporting cells (supplementary material Fig. S1C–E′). This suggests that Cdc42 is not involved in the regulation of the postmitotic state of the auditory sensory epithelial cells. Also cell survival was unaffected, based on the lack of staining for cleaved caspase-3, an apoptosis marker (data not shown). Further, expression of Prox1 in supporting cells (supplementary material Fig. S1D–E′), and Gfi1 (data not shown) and myosin 6 in hair cells were similar in control and mutant specimens (supplementary material Fig. S1F,G). Thus, cell fate decisions are not affected by *Cdc42* inactivation.

**Fig. 3.**
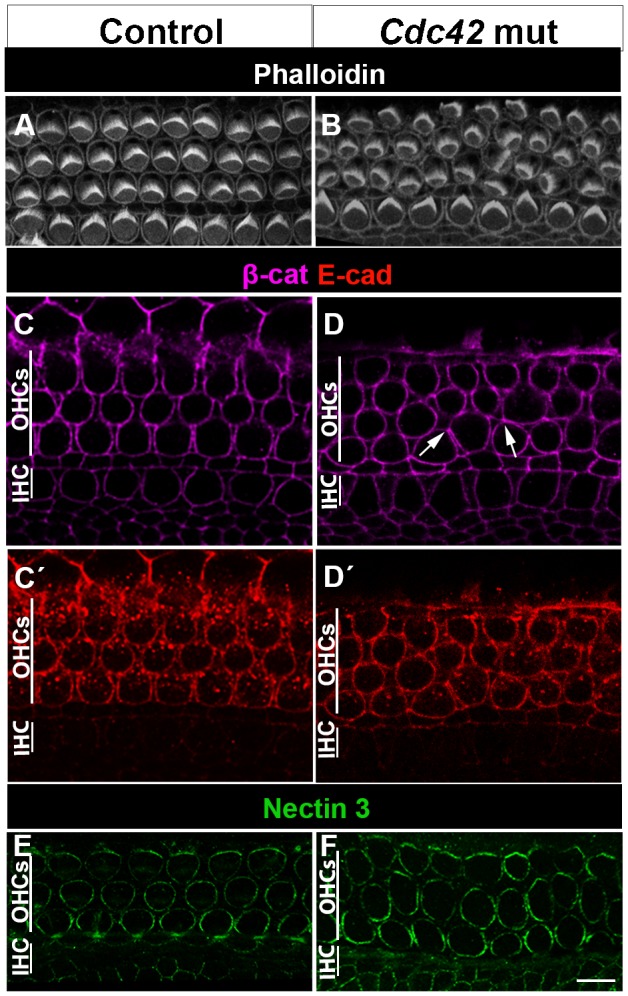
Cellular disorganization, but unaltered expression of adhesion proteins in the organ of Corti of *Cdc42^loxP/loxP^;Fgfr3-iCre-ER^T2^* mice at E18.5. (A,B) Phalloidin labeling shows the organization of OHCs into three rows in the control specimen. In the organ of Corti of the mutant mouse, misplacement of some OHCs in between the rows is seen. The organization of the row of unrecombined IHCs is unaltered. Both views are from the medial coil. (C–D′) Confocal views at the level of adherens junctions show comparable expression of β-catenin (C,D) and E-cadherin (C′,D′) in the two genotypes. Arrows in D point to atypical intercellular contacts. (E,F) Nectin 3 immunofluorescence is comparable in the contact sites between OHCs and Deiters' cells in both genotypes. Abbreviations: IHC, inner hair cell; OHC, outer hair cell; β-cat, β-catenin; E-cad, E-cadherin. Scale bar shown in F: A,B, 6 µm; C–F, 5 µm.

To study the possible involvement of Cdc42 in cellular patterning in the OC, whole mount specimens were labeled with phalloidin, marking F-actin. Cochlear specimens from control mice at E18.5 displayed precisely aligned hair cell rows, separated by supporting cell rows (Fig. 3A). In contrast, cochleas of the *Cdc42* mutant mice showed occasional misplacement of OHCs between the cell rows, creating direct contacts between OHCs, as opposed to normal OHC patterning with intervening supporting cells (Fig. 3B). These defects were seen in the medial coil of the cochlea and were mostly absent in the basal and apical coils. The unrecombined IHCs were properly aligned along the length of the cochlear duct of the mutant mice (Fig. 3A,B).

Proper alignment of the cells of the OC relies on dynamic remodeling of cell-cell contacts, a process dependent on adherens junctions (Chacon-Heszele et al., 2012). Therefore, we next studied whether changes in the expression of the adherens proteins E-cadherin, β-catenin as well as nectin 2 and nectin 3 could explain the OHC patterning defects. At E18.5, these proteins showed comparable expression in the lateral contact sites between OHCs and supporting cells (Deiters' cells) in both mutant and control specimens (Fig. 3C–F). Immunostaining also revealed comparable expression of the tight-junction-marker ZO-1 in OHCs of the two genotypes (data not shown). Thus, cellular disorganization triggered by *Cdc42* depletion is not caused by disturbed recruitment of these junctional components to the plasma membrane.

**Fig. 4.**
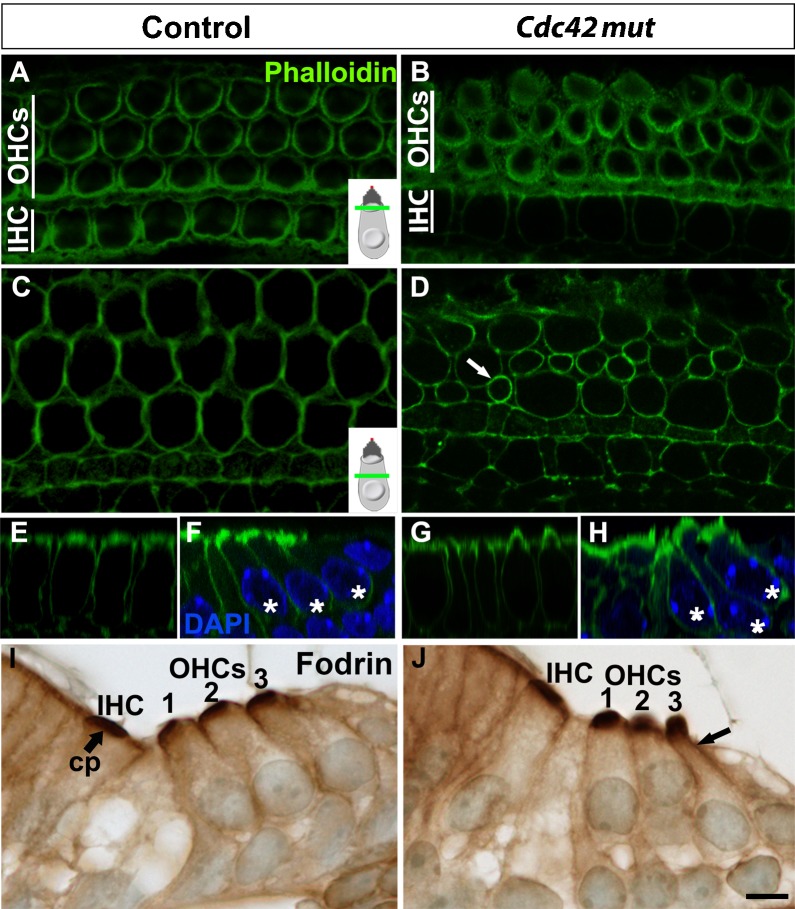
Cell shape changes in the organ of Corti of *Cdc42^loxP/loxP^;Fgfr3-iCre-ER^T2^* mice at E18.5. (A–H) Phalloidin-labeled whole mount specimens from the medial coil of the cochlea viewed under confocal microscopy. Views from the apical level of OHCs, just beneath the stereociliary bundles (A,B; the level defined in the inset), and from the level above the nuclei (C,D; the level defined in the inset). OHCs in the control specimen show uniform diameters, as opposed to OHCs in the mutant specimen. Arrow in D points to an OHC with a small, squeezed cell body. Variation in cell shapes in mutants can also be seen in y-z plane across one OHC row (E,G). In x-y (transverse) plane, DAPI-labeled OHC nuclei (asterisks) in the control cochlea are located at the same level, in contrast to the nuclei in the mutant cochlea (F,H). Note the squeezed cell body of the OHC with an abnormally positioned nucleus (H). (I,J) Immunohistochemistry on paraffin sections shows fodrin expression in the cuticular plates and lateral walls in the apical portion of OHCs of both genotypes. OHCs of the three rows are marked (1–3). In OHCs of mutant mice with a squeezed cell body, cortical fodrin expression extends basally (arrow). Abbreviations: OHC, outer hair cell; IHC, inner hair cell; cp, cuticular plate. Scale bar shown in J: A–J, 5 µm.

In addition to patterning defects, cochleas of mutant mice showed abnormally shaped OHCs, revealed in phalloidin-labeled whole mount specimens at birth (Fig. 4A–H). OHCs with squeezed morphology were found (Fig. 4E,G) and their nuclei were positioned at variable heights in the apico-basal axis of the sensory epithelium, in contrast to OHCs of control animals with uniformly positioned nuclei (Fig. 4F,H). Similar to patterning defects, OHCs with abnormal morphology were concentrated to the medial coil and were intermingled with OHCs with a normal morphology. To find out whether the atypical OHC shape was linked with changes in the expression of actin-interacting-proteins, paraffin sections were immunolabeled for alpha-fodrin (non-erythroid spectrin) that cross-links actin filaments. Fodrin is a constituent of the cuticular plate and cortical lattice of hair cells (Ylikoski et al., 1992). In agreement, control specimens showed fodrin expression in these regions in the apical portion of hair cells (Fig. 4I,J). In the *Cdc42* mutant mice, the cuticular plate staining was unaltered, but fodrin as well as phalloidin labeling in the cortex extended towards the basal side of the squeezed OHCs. This was best seen in top-down views showing F-actin accumulation at the most shrunken level of the atypical, flask-shaped OHCs (Fig. 4D). These data suggest that Cdc42 regulates the cortical actin cytoskeleton and that defects in this regulation lead to cell shape abnormalities.

### *Cdc42* inactivation leads to stereociliary bundle misorientation in outer hair cells

**Fig. 5.**
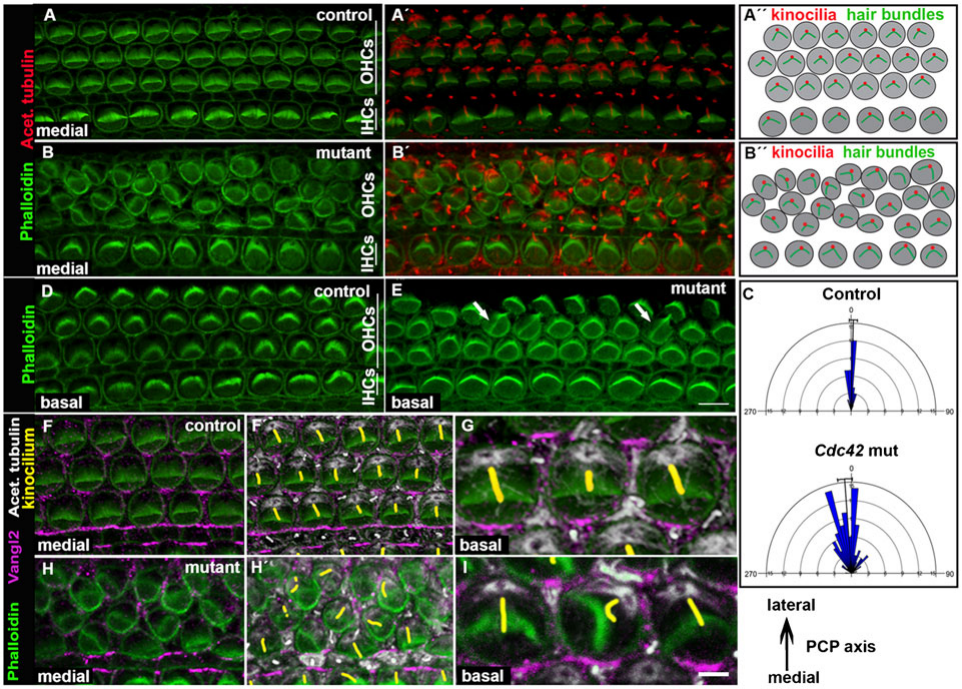
Misorientation of stereociliary bundles of outer hair cells of *Cdc42^loxP/loxP^;Fgfr3-iCre-ER^T2^* mice. Confocal images of whole mount specimens at E18.5. (A,A′) Shown by double-labeling, the medial coil of the control cochlea displays uniform orientation of phalloidin-labeled hair bundles and their laterally pointing vertices. The kinocilia, positive for acetylated tubulin, are located at vertices. (B,B′) The medial coil of the mutant cochlea shows several OHCs with misoriented bundles. (A″,B″) Schematic representations of hair bundle orientations. (C) Quantification (see materials and methods) shows the distribution of randomly oriented bundles in the medial coil of mutant cochleas. (D) Phalloidin labeling shows normal hair bundle orientation in the basal coil of the control cochlea. (E) The basal coil of the mutant specimen shows occasional OHCs with misoriented bundles (arrows), but hair cell rows are normally organized. (F–G) Triple-labeling for F-actin, Vangl2 and acetylated tubulin (kinocilia pseudocolored in yellow) shows Vangl2 expression in the contact sites between the OHC's medial wall and Deiters' cells in the control specimen. (H,H) In the medial coil of the mutant cochlea, Vangl2 expression pattern is influenced by cellular disorganization. (I) In the basal coil of the mutant cochlea where cellular disorganization is absent, Vangl2 expression domain is similar as in the control specimen (compare to Fig. 5G). Abbreviations: OHC, outer hair cell; IHC, inner hair cell. Scale bar shown in I: A–E, 6 µm; F–H′, 5 µm; G,I, 2 µm.

The read-out of PCP in the OC is the uniform orientation of hair bundles in the plane parallel to the epithelial surface. In cochlear whole mounts prepared from control and mutant mice at E18.5, phalloidin labeling was used to visualize the F-actin-rich stereocilia. Acetylated tubulin was used to mark kinocilia (Fig. 5A–E). In control specimens, hair bundles were uniformly oriented across the epithelium and kinocilia were positioned at the vertex of the bundles (Fig. 5A–A″). Cochleas of mutant mice showed OHC bundles with random orientation, vertices pointing to many directions, demonstrating an abnormal planar polarity phenotype. Unrecombined IHCs showed unaltered bundle orientation (Fig. 5A–E). Similar to patterning and shape abnormalities, bundle orientation defects were concentrated, but not restricted to the medial coil (Fig. 5B–B″). Quantification performed in the medial coil revealed a clear difference in OHC bundle orientations between mutant and control specimens (Fig. 5C). In the medial coil, the same OHC often displayed all abnormalities. In contrast, the basal coil showed OHCs with misoriented bundles lacking patterning and shape defects (Fig. 5D,E). Thus, bundle misorientation can occur independently of the defects at the level of the cell body. This was also found in experiments where induction of recombination was shifted from E13.5 and E14.5 to E15.5 and E16.5, assuming that this later onset of *Cdc42* inactivation does not cause patterning defects in the medial coil. Despite the fact that there was a lack of patterning and shape defects, OHCs still showed a planar polarity defect when analyzed at E18.5 (data not shown).

To quantitate the relationship between disturbances in stereociliary bundle orientation and shape of OHCs, we measured cell diameters at the level of adherens junctions at E18.5 and correlated these values to bundle orientation (supplementary material Fig. S2). Orientation of hair bundles did not correlate with the diameter of cell bodies. Thus, these two phenotypical changes of OHCs of the *Cdc42* mutant mice can occur independently of each other.

### Expression of the core PCP components is not affected by *Cdc42* inactivation

Based on the altered PCP phenotype of OHCs of the *Cdc42* mutant mice, we next examined the possible link between Cdc42 and the Wnt/PCP pathway (Fig. 5F–I). Cochlear surface specimens were prepared at E18.5 and stained for Vang-like 2 (Vangl2) (Montcouquiol et al., 2003) and Frizzled 6 (Wang et al., 2006), the core components of the PCP pathway. In control animals, Vangl2 was expressed at the level of adherens junctions, in contact sites between supporting cells and the medial side of the OHC plasma membranes (Fig. 5F,F′,G). By high resolution microscopy, this expression has been localized to supporting cells (Montcouquiol et al., 2006; Giese et al., 2012). The medial coil of mutant mice showed unaltered Vangl2 expression (Fig. 5H,H′). To exclude the effects of cellular disorganization on Vangl2 expression, we focused on the basal coil where OHCs displayed bundle disturbances exclusively. Triple-labeling for F-actin, acetylated tubulin and Vangl2 revealed unaltered Vangl2 expression, despite hair bundle abnormalities (Fig. 5I). Similarly, membraneous Frizzled 6 expression on the medial side of the OHC apex (Wang et al., 2006) was unaltered in mutant animals (data not shown). These findings exclude a direct relationship between Cdc42 and Wnt signaling.

### *Cdc42* inactivation leads to stereociliary bundle dysmorphology and kinocilia mispositioning in outer hair cells

**Fig. 6.**
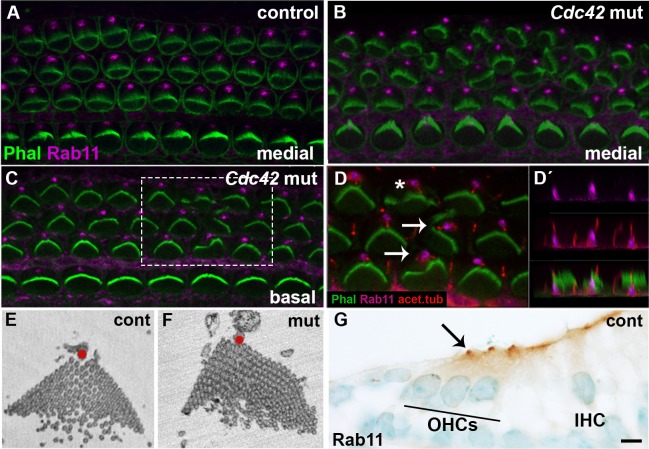
Disturbances in stereociliary bundle morphology, but maintained Rab11a expression in outer hair cells of *Cdc42^loxP/loxP^;Fgfr3-iCre-ER^T2^* mice. (A) Confocal view of a whole mount specimen from the medial part of the control cochlea at E18.5 shows Rab11 expression close to the vertices of phalloidin-labeled hair bundles. (B,C) Both in the medial and basal coil of the mutant cochlea, Rab11 is expressed similarly as in the control specimen. (D,D′) Higher magnification view of the boxed area in C shows partial colocalization of acetylated tubulin, a kinocilium marker, and Rab11 at the base of kinocilia. This colocalization is best revealed in transverse plane (D′). A part of OHCs in this mutant specimen show dysmorphic bundles accompanied by off-centered kinocilia (arrows). Note that bundle dysmorphology exists also in normally oriented OHCs (asterisk). (E,F) SBEM images of OHC stereociliary bundles of control and mutant cochleas at E18.5. In the control specimen, kinocilium (red dot) is located at the vertex of the V-shaped hair bundle. In the mutant specimen, kinocilium is misplaced and the bundle is dysmorphic, with no clear vertex. (G) Also transverse sections immunohistochemically stained for Rab11 demonstrate expression in the apex of OHCs. Abbreviations: OHC, outer hair cell; IHC, inner hair cell. Scale bar shown in G: A–D″, G, 5 µm; E,F, 1 µm.

In OHCs of the mutant mice, phalloidin labeling demonstrated dysmorphic hair bundles. In most cases, bundles were misoriented as well (Fig. 6A–D), but dysmorphic bundles lacking an orientation defect were also found (Fig. 6C,D). Abnormal bundle morphology, a read-out of altered hair cell intrinsic polarity (Sipe and Lu, 2011; Fukuda et al., 2014), was manifested as flat, wavy or inverted shape, often with no clear vertex. Importantly, bundle dysmorphology was often associated with abnormal positioning of the kinocilium relative to the bundle, as revealed by using acetylated tubulin as a kinocilium marker (Fig. 6C–D″). These findings were ultrastructurally confirmed by SBEM (Fig. 6E,F).

Prior studies have shown that normal hair bundle morphology depends on proper development and migration of the kinocilium/basal body complex (Ross et al., 2005; Jones et al., 2008; Cui et al., 2011; Sipe and Lu, 2011). During late-embryogenesis, this complex starts to develop at the center of the hair cell's surface and it migrates towards the lateral cell periphery. In parallel, surrounding microvilli elongate to form stereocilia of graded heights. Around birth, the immature bundle is V-shaped with the kinocilium at the vertex, next to the tallest stereocilia (Jones and Chen 2008). Interestingly, Cdc42 has been shown to be crucial for the assembly of primary cilia in other cell types (Zuo et al., 2011; He et al., 2012; Choi et al., 2013). Based on these prior data, we next asked whether *Cdc42* depletion influences OHC planar polarity by regulating the assembly or migration of the kinocilium/basal body.

Cochleas at E18.5 were stained for acetylated tubulin marking the axoneme of kinocilium, for γ-tubulin marking centrioles and for the GTPase Rab11a that has been shown to mark the basal region of primary cilia and to be critical for primary ciliogenesis (Knödler et al., 2010; Westlake et al., 2011; He et al., 2012). Cdc42 has been proposed to be involved in the process of primary ciliogenesis by targeting the exocyst and Rab11a-Rabin-Rab8 trafficking complexes (Zuo et al., 2011; He et al., 2012). We found strong, condensed Rab11a expression in the base of hair cell kinocilia, similar as reported in primary cilia of other cell types. This staining was found both in control and mutant specimens (Fig. 6A–D′,G). Also, acetylated tubulin expression in the axoneme was comparable in the two genotypes (Fig. 6A–D′), similar to the localization of γ-tubulin-positive centrioles relative to the bundle (data not shown). These results suggest that the loss of Cdc42 does not cause impaired assembly of the kinocilium.

At E18.5, double-labeling for F-actin and acetylated tubulin revealed that the center-to-periphery migration of the kinocilium/basal body complex had taken place in OHCs of both control and mutant mice. However, kinocilia of mutant animals showed deviation from their final, lateral positioning (Fig. 6D). These findings suggest that Cdc42 regulates planar cell polarity at stages after primary kinocilium/basal body migration (Lepelletier et al., 2013).

### *Cdc42* inactivation impairs microtubule organization and alters aPKC expression in the apices of outer hair cells

**Fig. 7.**
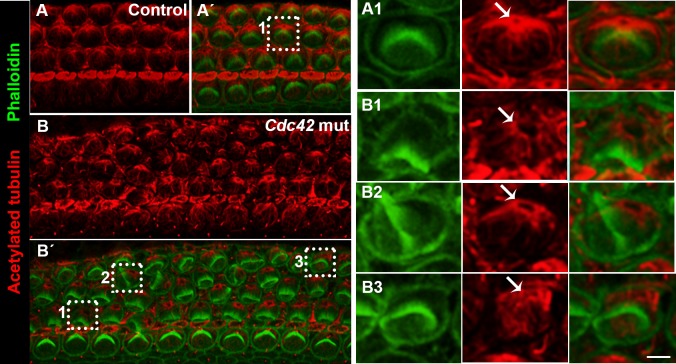
Disorganized apical microtubule network in outer hair cells of *Cdc42^loxP/loxP^;Fgfr3-iCre-ER^T2^* mice. Confocal views of cochlear whole mount specimens dissected from the medial coil of control and mutant mice at E18.5. (A–B′) Specimens double-labeled for acetylated tubulin, marking microtubules and basal body, and for phalloidin that labels the hair bundle. Z-stacks cover the level from the basal body to the apicalmost astral microtubules of OHCs. Boxed OHCs in (A′,B′) are shown in higher magnification in A1,B1,B2,B3. (A1) Control specimen shows the localization of the basal body (arrow) close to the vertex of hair bundle and ordered radiation of microtubules at the OHC surface. (B1–B3) In the mutant specimen, the microtubule network is rotated with respect to hair bundle misorientation and microtubules around the basal body are disorganized (B1). Some OHCs show complete loss of the ordered microtubular radiation (B3). Scale bar shown in B3: A–B′, 5 µm; A1–B3, 2 µm.

In the apices of epithelial cells, astral microtubules radiate from the basal body and are captured by plus-ends to the cell cortex. Pulling forces provided by microtubules are essential for the migration of the basal body and primary cilium and for docking of these structures to their final position (Tang and Marshall, 2012). Recent studies on cochlear hair cells have linked abnormal positioning of the kinocilium to disorganized cell-surface microtubule network (Ezan et al., 2013; Sipe et al., 2013). Compared to control specimens (Fig. 7A–A1), acetylated tubulin-staining at E18.5 showed disorganized microtubules at the OHC surface in the *Cdc42* mutant mice (Fig. 7B–B3). Both microtubules around the basal body and those radiating at the cell surface were abnormal. Further, both dysmorphology of the hair bundle and abnormal position of the basal body/kinocilium correlated with microtubule disorganization, a finding clearly seen in high magnification views (Fig. 7B1–B3). These results suggest that altered planar polarity of OHCs is mediated by microtubular disturbances.

**Fig. 8.**
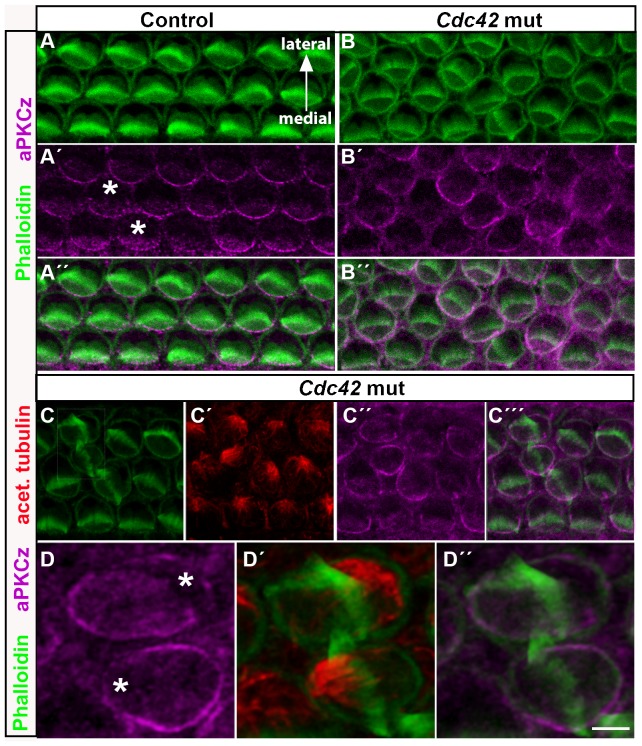
Altered aPKCζ expression in outer hair cells of *Cdc42^loxP/loxP^;Fgfr3-iCre-ER^T2^* mice. Confocal images of whole mount specimens dissected from the medial cochlear coil of control and mutant mice at E18.5 and labeled for F-actin, acetylated tubulin and aPKCζ. All images are shown in medial-lateral orientation, as indicated in (A). (A–A″) In the control specimen, aPKC is expressed in the cortical and, weaker, in the cytoplasmic domain at the OHC surface, medial to the hair bundle. Asterisks mark the lateral, aPKC-negative domain. (B–B″) In the mutant specimen, both cortical and cytoplasmic aPKC expression domains are rotated with respect to hair bundle misorientation. (C–C′″) In the mutant specimen, triple-labeling for acetylated tubulin, phalloidin and aPKC shows that the rotation of aPKC expression parallels the rotation of the astral microtubule network and hair bundle misorientation. (D–D″) High magnification views of two OHCs with abnormal bundles. The OHC below has a completely turned bundle. Asterisks mark the concise aPKC-free area in these cells (compare to control OHCs in Fig. 8A′). Scale bar shown in D″: A–C′″, 5 µm; D–D″, 2 µm.

In several cell types, Cdc42 regulates polarization through aPKC, and this signaling has been shown to control cell polarity through the regulation of microtubule dynamics (Etienne-Manneville, 2004; Etienne-Manneville et al., 2005). Therefore, we next focused on aPKC expression at the OHC's apical surface. It has been previously shown that the aPKC isoform λ/ι is not expressed in the OC around birth (P2), but becomes upregulated a few days thereafter (Anttonen et al., 2012). We found that the aPKCζ isoform is expressed in the hair cell apex during late-embryogenesis. Most prominent expression was found in the medial plasma membrane, opposite to the vertex of the hair bundle (Fig. 8A–A″). Weaker cytoplasmic expression was seen on the medial side at the cell surface (Fig. 8A–A″). These findings are consistent with prior data revealing that the hair bundle is positioned at the interface of aPKC-positive and -negative domains at the hair cell apex (Ezan et al., 2013; Tarchini et al., 2013). Based on triple-labeling with phalloidin and antibodies against ZO-1 and aPKCζ or phospho-aPKC (marking activated kinase), aPKCζ (data not shown) and phospho-aPKC (supplementary material Fig. S3) were localized to the level of tight junctions of OHCs.

In OHCs of the *Cdc42* mutant mice, the membraneous aPKC expression domain was altered and it corresponded to the degree of abnormal rotation of the hair bundle (Fig. 8B–D″). This is most clearly seen in the high magnification view of an OHC with a completely turned bundle (Fig. 8D). Further, in OHCs where the bundle and kinocilium were abnormally positioned at the very lateral edge of the cell surface, membraneous and cytoplasmic aPKC expression was extended to this lateral side (Fig. 8D). Based on triple-labeling with phalloidin and antibodies against aPKCζ and acetylated tubulin, microtubule disorganization was coupled with hair bundle defects and altered aPKC expression (Fig. 8C–C′″). These results suggest that Cdc42 and its putative downstream effector, aPKC, regulate the establishment of hair cell planar polarity via the microtubule cytoskeleton.

### Planar polarity defect is maintained and stereociliogenesis impaired in outer hair cells of the *Cdc42* mutant mice early postnatally

**Fig. 9.**
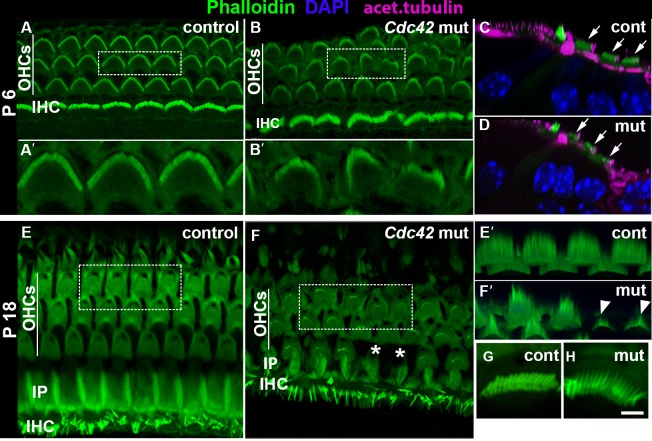
Impaired stereociliogenesis in outer hair cells of *Cdc42^loxP/loxP^;Fgfr3-iCre-ER^T2^* mice postnatally. Confocal views of whole mount specimens from the medial coil of the cochlea. Boxes in A,B,E,F are enlarged in A',B',E',F', respectively. (A,A′) Phalloidin labeling shows the typical hair bundle morphology in the control specimen at P6. (B,B′) In the mutant cochlea at P6, all OHCs with stereociliary bundles are present, but some bundles are misoriented and have a fragmented or stunted appearance. (C,D) Degeneration of OHC bundles is also seen in transverse views where DAPI labels nuclei and acetylated tubulin marks kinocilia and microtubules. Arrows point to outer hair cells. (E,E′) Phalloidin labeling reveals the normal cellular organization and hair bundle morphology in the control specimen at P18. (F,F′) In the mutant cochlea, scattered OHC loss is seen (asterisks in F). Also, OHC bundles are fragmented and often composed of very short stereocilia (arrowheads in F′). (G,H) Unrecombined IHCs of mutants show stereociliary bundles comparable to controls. Abbreviations: OHCs, outer hair cells; IHC, inner hair cell; IP, inner pillar cell. Scale bar shown in H: A–D, 5 µm; E′,F′ 3 µm; A′,B′,G,H, 2 µm.

To study the fate of OHCs with abnormal apical polarity, cochleas of the *Cdc42^loxP/loxP^;Fgfr3-iCre-ER^T2^* mice (tamoxifen administration at E13.5 and E14.5) were analyzed at P6. Phalloidin labeling showed a normal complement of auditory hair cells in these animals, demonstrating that *Cdc42* inactivation does not abrogate the survival of juvenile hair cells. OHCs with misoriented and dysmorphic stereociliary bundles were maintained in these cochleas. Acetylated tubulin immunostaining revealed the presence of the kinocilium in OHCs of both mutant and control mice. Notably, the staircase pattern of stereociliary bundles was in many cases disrupted. Bundles also appeared fragmented, suggesting that stereociliogenesis itself was impaired (Fig. 9A–D), consistent with strong Cdc42 expression in stereocilia (Fig. 2D). To confirm the involvement of Cdc42 in stereociliogenesis, P18 mutant mice were analyzed. In contrast to OHCs of control specimens that showed long and precisely arranged stereocilia, OHCs of the mutant mice had fragmented hair bundles consisting of stereocilia of variable lengths, some being very short (Fig. 9E,F). The unrecombined IHCs with normal bundles served as controls (Fig. 9G,H). Interestingly, at P18, besides the defects in OHC stereociliogenesis, scattered OHC loss was observed (Fig. 9F). Together, in addition to the control of hair cell patterning, shape and planar polarity, these results give evidence that Cdc42 regulates postnatal stereociliogenesis.

## DISCUSSION

Cdc42 is involved in many cellular processes by regulating cytoskeletal and junctional dynamics and, thereby, it critically influences apical cell polarization (Etienne-Manneville, 2004). *In vivo* studies in the embryonic mouse have revealed the importance of Cdc42 in the establishment of apico-basal polarity of proliferating progenitor cells (Cappello et al., 2006; Kesavan et al., 2009; Melendez et al., 2013). Cdc42 can also regulate later developmental events, as shown in the cochlea shortly after birth (Anttonen et al., 2012). In that study, a conditional, inducible *Cdc42* inactivation strategy was used to obtain a mutant mouse model where embryonic development was bypassed. In the present study, *Cdc42* is acutely inactivated in the late-embryonic OC, shortly after terminal mitoses when sensory epithelial cells start to differentiate. We show the versatile role of Cdc42 in structural differentiation of hair cells. We focus the discussion on its role in OHC's apical polarization and take into account the fact that several molecules involved in the establishment of apical polarity are known to converge on Cdc42 signaling.

The CE process of cell movements consists of directed migration and polarized rearrangement of cells (Wallingford et al., 2002). CE has an important role during cochlear development. Between E14.5 and E18.5 in the mouse, cells of the OC become arranged into precise rows by means of CE, leading to thinning and lengthening of the cochlear duct (McKenzie et al., 2004; Wang et al., 2005). Interestingly, Cdc42 has been shown to regulate CE during gastrulation in non-mammalian species (Choi and Han, 2002; Yeh et al., 2011). It is likely that the possible role of Cdc42 in CE in the cochlea could be revealed with our gene inactivation strategy, not only because of the timing of onset of *Cdc42* inactivation (between E13.5 and E14.5), but also because the inactivation took place in OHCs and two types of supporting cells, the pillar and Deiters' cells, constituting a broad region of the cochlear epithelium. We did not see changes in dimensions of the OC of *the Cdc42^loxP/loxP^;Fgfr3-iCre-ER^T2^* mice, excluding a primary role for Cdc42 in the regulation of global CE events in the cochlea. This suggestion is supported by unaltered expression of the adherens junction components E-cadherin, β-catenin and nectins in the OC of the mutant mice, taking into account prior data that E-cadherin and N-cadherin expressions are abolished in hair cells when CE is impaired (Chacon-Heszele et al., 2012). However, we cannot exclude the possibility of a fine-grained function of Cdc42 in CE in the cochlea.

OHCs of the *Cdc42* mutant mice showed misoriented and dysmorphic stereociliary bundles together with mispositioned kinocilia, the characteristic features of an abnormal hair cell intrinsic polarity phenotype. The kinocilium is essential for driving proper hair bundle morphogenesis (Ross et al., 2005; Sipe and Lu, 2011; Cui et al., 2011). In the mutant mice, we did not find defects in kinocilium assembly or primary migration of the kinocilium/basal body complex to the lateral side of OHC surface. The basal body serves as a microtubule-organizing center (MTOC) for cytoplasmic microtubules. Microtubules are connected to the cell cortex and have a crucial role in defining cell shape and polarity (Li and Gundersen, 2008). Interestingly, during the establishment of planar hair cell polarity, defects in cell-surface microtubules have been shown to lead to kinocilium/basal body mispositioning and improper bundle morphology and orientation (Sipe and Lu, 2011; Sipe et al., 2013, Ezan et al., 2013). We found that the microtubule network at the apex of *Cdc42*-depleted OHCs was disorganized, suggesting that the abnormal hair cell intrinsic polarity phenotype could result from perturbed microtubule dynamics. This suggestion is supported by our localization data, revealing Cdc42 expression in the pericentrosomal region, the region from where microtubules radiate (Mogensen et al., 1997). One candidate factor mediating the effect of Cdc42 in this region is dynein, a member of the microtubule motor protein complex. Similar to Cdc42, dynein has been localized in developing hair cells to the region around the centrosome and has been suggested to be involved in basal body positioning (Sipe et al., 2013). Interestingly, in other cellular models, Cdc42/dynein interaction orientates microtubules radiating from the MTOC (Palazzo et al., 2001).

In addition to the expression of Cdc42 in the basal body region, we localized Cdc42 to the medial side of the OHC's apical surface, so that its expression flanked the hair bundle position. Such an expression pattern of Cdc42 was comparable to that of aPKCζ, a well-known Cdc42 effector. Furthermore, an interaction between these proteins was suggested by altered aPKC expression in OHCs of the mutant mice. Importantly, the altered aPKC expression domain at the hair cell apex correlated with abnormal bundle position along the medio-lateral axis. Thus, Cdc42 and aPKC appear to be part of the machinery establishing compartmentalization of the hair cell apex and thereby planar cell polarity. This suggestion is consistent with recent reports on the role of aPKC in developing hair cells (Ezan et al., 2013; Tarchini et al., 2013). Our suggestion is also in agreement with the data on migrating astrocytes in which the Cdc42/aPKC complex promotes interactions between the microtubule plus-ends and lateral cell cortex proteins (Etienne-Manneville and Hall, 2001). Thus, in addition to the role of Cdc42 in the pericentrosomal region, Cdc42 and aPKC might regulate the establishment of hair cell planar polarity through microtubules and thereby might couple hair bundle development to the cell cortex.

In the apices of OHCs of the *Cdc42* mutant mice, disturbances in the hair bundle and kinocilium correlated with the impaired network of astral microtubules and with the altered cortical expression of aPKC, the two well-known components of the apical cell polarity machinery. The obvious question is whether the abnormalities in the OHC apex are coupled with the defects in patterning and shapes of OHC bodies also found in the mutant mice. OHCs with only abnormal hair cell intrinsic polarity were found, suggesting that this altered phenotype can arise independently at the hair cell surface. However, bundle and kinocilium defects were most pronounced in OHCs showing disturbances in cellular patterning as well. This suggests that the establishment of hair cell planar polarity is influenced by lateral cell compartments and by contacts between hair cells and supporting cells. This suggestion is also in line with our findings that Cdc42 is expressed in junctions between these cell types and with the fact that *Cdc42* was inactivated in both cell types in our mutant mouse model. In agreement, it has been shown that inactivation of nectin 3, an adherens protein of supporting cells, triggers not only cellular mispatterning in the OC, but also disturbances in orientation and morphology of hair bundles and positioning of kinocilia (Fukuda et al., 2014). Our expression analysis excluded the possibility of nectins functioning as downstream targets of Cdc42. However, an intriguing possibility remains that Cdc42 is an effector of nectins in hair cells, similar as shown in other types of epithelial cells (Fukuhara et al., 2004).

The Rho GTPase Rac1 regulates cellular organization and hair cell polarity in the late-embryonic OC (Grimsley-Myers et al., 2009). These processes are also controlled by Lis1, a regulator of microtubule dynamics and a potential upstream regulator of both Cdc42 and Rac1 (Kholmanskikh et al., 2006; Sipe et al., 2013). The defects in OHCs of the *Cdc42* mutant mice resembled the phenotypes of *Rac1* and *Lis1* mutant mice (Grimsley-Myers et al., 2009; Sipe et al., 2013). This may indicate functional compensation between the two Rho GTPases, a possibility that would explain why defects were not manifested in all recombined OHCs in the *Cdc42* mutant mice. It has been shown that the position of the kinocilium/basal body is not yet stabilized at birth (Sipe et al., 2013). Furthermore, hair cell planar polarity is refined postnatally (Copley et al., 2013). We found that, in the neonatal *Cdc42* mutant mice, the kinocilium/basal body complex was maintained on the lateral side of the OHC surface, indicating that Cdc42 is not a primary regulator of sustained planar polarity, as opposed to Lis1 (Sipe et al., 2013). Further, as misoriented hair bundles were maintained in these animals, the machinery responsible for polarity refinement (Copley et al., 2013) cannot overcome the polarity defects triggered by *Cdc42* inactivation at late-embryogenesis.

In conclusion, consistent with the involvement of Cdc42 in multiple signaling cascades and in the regulation of cytoskeletal dynamics, the present results show that Cdc42 regulates various aspects of hair cell differentiation. Our results suggest that Cdc42 is involved in OHC stereociliogenesis early postnatally, likely through the regulation of actin dynamics (Ridley, 2006). Our data also demonstrate the role of Cdc42 in cellular patterning and in mediating planar polarity of embryonic OHCs. Together, these data show that Cdc42 is important for the establishment of proper hearing function.

## Supplementary Material

Supplementary Material
