# Supplementary material for: The Rho GTPase Cdc42 regulates hair cell planar polarity and cellular patterning in the developing cochlea
Source: Biol Open. 2015 Mar 13;4(4):516–26. doi: 10.1242/bio.20149753 (PMC4400594; doi:10.1242/bio.20149753)
Supplement: Supplementary Material [file supp_4_4_516__index.html]

The Rho GTPase Cdc42 regulates hair cell planar polarity and cellular patterning in the developing cochlea — The Rho GTPase Cdc42 regulates hair cell planar polarity and cellular patterning in the developing cochlea — Supplementary Material 

# The Rho GTPase Cdc42 regulates hair cell planar polarity and cellular patterning in the developing cochlea

## bio.20149753 Supplementary Material

**Files in this Data Supplement:**

- Supplementary Material - Anna Kirjavainen et al. doi: 10.1242/bio.20149753
