# Supplementary material for: The Rho GTPase Cdc42 regulates hair cell planar polarity and cellular patterning in the developing cochlea
Source: Biol Open. 2015 Mar 13;4(4):516–26. doi: 10.1242/bio.20149753 (PMC4400594; doi:10.1242/bio.20149753)
Supplement: Supplementary Material [file supp_bio.20149753_bio.20149753-s1.pdf]

Biology Open (2015) 000, 1–11 doi:10.1242/bio.20149753

Supplementary Material

Anna Kirjavainen et al. doi: 10.1242/bio.20149753

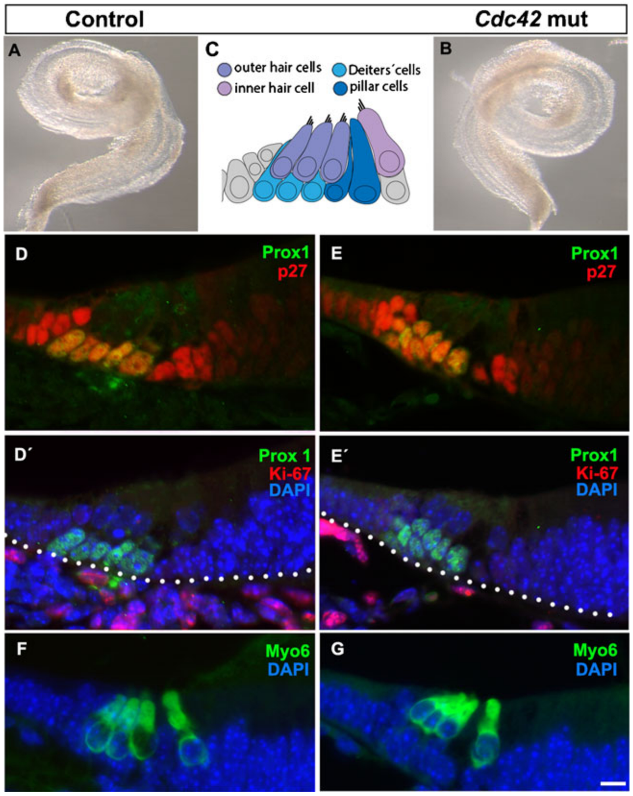

Fig. S1. Unaltered global structure of the cochlea. Unaltered expression of the cell cycle and cell type-specific markers in the organ of Corti of *Cdc42$^{loxP/loxP}$;Fgfr3-iCre-ER^{T2}* mice at E18.5. (A,B) The dimensions of the cochlear duct are comparable in control and mutant mice, shown in whole mount preparations. (C) Schematic representation of cellular organization in the organ of Corti. (D,E) Double-immunofluorescence on sections shows strong p27^{Kip1} expression in supporting cells surrounding hair cells both in control (D) and mutant cochleas (E). Dotted line marks the basilar membrane underneath the organ of Corti. (D′,E′) Both genotypes show the absence of Ki-67 expression in the organ of Corti and maintained Prox1 expression in supporting cells (Deiters' and pillar cells). (F,G) Myosin 6 is expressed in hair cells of both types of animals. DAPI marks nuclei. Abbreviation: Myo6, Myosin 6. Scale bar shown in G: A,B, 40 µm; D–G, 5 µm.

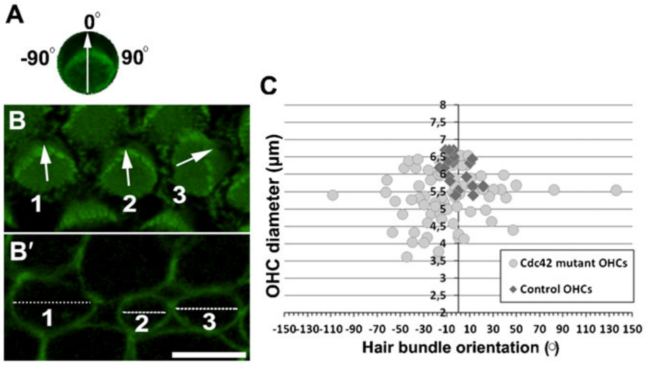

Fig. S2. Quantification of the relationship between the cell body diameter and stereociliary bundle orientation of outer hair cells of Cdc42$^{loxP/loxP}$;Fgfr3-iCre-ER^{T2} mice at E18.5. (A–B′) Hair bundle orientation, determined as shown in phalloidin-labeled views (A,B), and cell body diameter, measured at the level of adherens junctions, of the same cells (B′; cells 1–3). (C) Scatter plot drawn based on regression analysis shows that hair cell diameter and hair bundle orientation do not have a causal relationship ($R^2 = 0{,}014$, $P=0.3$). In controls, cell diameters and hair bundle orientations are distributed in a condensed manner along the x and y axes Scale bar: 5 µm.

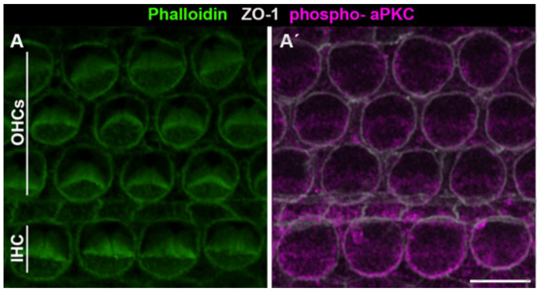

Fig. S3. aPKC is expressed at the level of tight junctions in hair cells. (A,A′) A cochlear whole mount specimen prepared from a control mouse at E18.5 and triple-labeled with phalloidin and antibodies against phospho-aPKC and ZO-1. Abbreviations: IHC, inner hair cell; OHCs, outer hair cells. Scale bar: 5 µm.
